# Niche adaptation of particle-associated ammonia-oxidizing archaea sustains nitrification under marine deoxygenation

**DOI:** 10.3389/fmicb.2026.1773718

**Published:** 2026-03-12

**Authors:** Li Li, Duo Zhao, Rui Du, Kai Tang, Yao Zhang

**Affiliations:** State Key Laboratory of Marine Environmental Science and College of Ocean and Earth Sciences, Xiamen University, Xiamen, China

**Keywords:** ammonia-oxidizing archaea, key functional genes, marine deoxygenation, metagenome-assembled genomes, nitrification rate, nitrogen cycling, particle-associated niche adaptation

## Abstract

Marine deoxygenation is restructuring coastal microbial niches and metabolic networks, with cascading effects on biogeochemical cycles, a key component of which is the nitrogen cycle. Particles constitute a critical ecological interface that mediates microbial niche partitioning and oxygen-sensitive balance between nitrogen loss and retention in deoxygenating coastal waters. However, the niche-dependent metabolic partitioning of microbial communities and its influence on the nitrogen cycle under deoxygenation remains poorly constrained. We conducted a 22-day field investigation of the deoxygenated water column off the Zhoushan coast, China, combining temporal ^15^N-tracer-based nitrification rate measurements with size-fractionated metagenomic sequencing during the day of the most severe bottom-water oxygen depletion. Our data revealed a nitrification hotspot in the low-oxygen waters below the pycnocline, with persistently elevated rates and an enriched abundance of ammonia-oxidizing archaea (AOA) and nitrite-oxidizing bacteria. Notably, particle-associated AOA exhibited significantly enriched genomic potential for coupled nitrogen cycling and carbon fixation, while the dominant groups adapted to low-oxygen particles via distinct metabolic strategies. *Nitrosomarinus*-like AOA exhibited higher gene counts (*amoA*-normalized) for ammonia (*amt*) and high-affinity phosphate (*pst*) transporters, whereas their Water column group A-like counterparts were enriched in low-affinity phosphate transporters (*pit*). Urease gene enrichment in both major AOA clades implicates urea as an ecologically relevant alternative nitrogen source for ammonia acquisition in coastal waters. Furthermore, particle-associated AOA may couple nitrite production and consumption via co-enriched ammonium monooxygenase (*amoA*) and nitrite reductase (*nirK*), potentially increasing nitrogen loss through local nitrite utilization. Collectively, our findings demonstrate that differential adaptation across clades underpins the pivotal role of AOA in nitrogen cycling under deoxygenation.

## 1 Introduction

The loss of dissolved oxygen (DO) in the oceans, known as deoxygenation, is one of the most significant threats to marine ecosystems (Keeling et al., 2010; Rabalais et al., 2014; Levin and Breitburg, 2015; Levin, 2018). Recent studies have documented increasing occurrences of seasonal deoxygenation in coastal ecosystems globally (Guo et al., 2020; Sheng et al., 2024). Deoxygenation in estuarine and coastal zones is primarily driven by anthropogenic eutrophication, which enhances remineralization, and can be further intensified by seasonal water column stratification that suppresses vertical mixing (Frölicher et al., 2009; Breitburg et al., 2018; Oschlies et al., 2018; Zhang et al., 2025). The Yangtze River estuary and adjacent East China Sea are representative examples of intensifying seasonal bottom-water hypoxia (Zhang et al., 2022; Sheng et al., 2024). Deoxygenation-induced low-oxygen zones are widely recognized as biogeochemical hotspots, hosting diverse microbially mediated nitrogen transformation processes (Beman et al., 2013; Bristow et al., 2013; Sun et al., 2017). Among these, nitrification, the conversion of reduced inorganic nitrogen to nitrate mediated by ammonia-oxidizing archaea (AOA) and nitrite-oxidizing bacteria (NOB), serves as a critical bridge between nitrogen regeneration (e.g., remineralization) and nitrogen loss pathways (e.g., denitrification) (Lam et al., 2007; Ward et al., 2009; Peng et al., 2016; Haas et al., 2021; Tang et al., 2023). Even at extremely low DO concentrations (e.g., nanomolar levels or below the detection limit), AOA can sustain ammonia oxidation activity (Bristow et al., 2016; Sollai et al., 2019) through their unique capacity to enzymatically generate trace oxygen via nitric oxide dismutation, as demonstrated in *Nitrosopumilus maritimus* SCM1 (Kraft et al., 2022).

Particles in low-oxygen zones create distinct microhabitats that strongly modulate nitrogen cycling dynamics (Klawonn et al., 2015; Wan et al., 2023a; Wan et al., 2023b). For instance, enhanced NH_4_^+^ regeneration via remineralization within particle-associated microenvironments can facilitate nitrification in these localized niches (Ciccarese et al., 2023; Hemsley et al., 2023; Ma et al., 2021). Consistently, nitrifiers are frequently enriched on particles, with their distributions and activities further characterized by a distinct particle-size dependence (Xia et al., 2009; Hou et al., 2018; Ma et al., 2021). Moreover, size-fractionated ^15^N-tracer incubation experiments indicate that particle-associated nitrification rates can exceed those of free-living counterparts and may contribute substantially to bulk nitrification in such ecosystems (Zheng et al., 2017). Although particle-associated nitrification is recognized in estuarine-coastal systems, its magnitude varies substantially across ecosystems and seasons. More importantly, quantitative linkages between nitrification activity and the specific traits of particle-associated nitrifier communities remain poorly constrained, especially in the context of dynamic coastal hypoxia (Hsiao et al., 2014; Zheng et al., 2017; Kache et al., 2021). It is notable that much of our mechanistic insight comes from stable open-ocean oxygen minimum zones (OMZs), whereas coastal hypoxia occurs in shallower, particle-laden waters with highly variable oxygen regimes, a combination likely to select for unique nitrifier communities and functional traits (Rabalais et al., 2014; Bristow et al., 2016; Breitburg et al., 2018). Understanding the niche partitioning of nitrifiers between particle-associated (PA) and free-living (FL) lifestyles in coastal hypoxia, as well as their metabolic versatility under fluctuating oxygen conditions, is essential for predicting how coastal nitrogen cycling reconfigures under deoxygenation.

Here, we selected Dongji Island in the East China Sea, a natural laboratory featuring a 5-km^2^ submarine basin that undergoes pronounced deoxygenation, to investigate the ecology of nitrifying microbial communities in fluctuating oxygen regimes. By combining ^15^N stable isotope tracer measurements of nitrification rates with size-fractionated metagenomic and genome-resolved analyses, we aimed to (1) quantify how nitrification rates varied across the vertical oxygen gradient; (2) resolve spatial variations in FL and PA microbial community structure along the oxygen gradient; (3) identify dominant AOA clades driving nitrification and quantify the relative abundances of key functional genes; and (4) elucidate the metabolic versatility of AOA and their niche-adaptive strategies for particulate matter colonization.

## 2 Materials and methods

### 2.1 Sample collection and environmental parameter measurements

Seawater samples were collected over a 22-day period from July 14 to August 4, 2022, on six occasions from three depths (5, 25, and 50 m) at Station C4 (30.08°N, 122.44°E) adjacent to Dongji Island. These samples were used in incubation experiments with N-isotope tracers to determine the rates of ammonia and nitrite oxidation. In addition, approximately 30 L of seawater was initially pre-filtered through a 20-μm mesh, followed by sequential filtration using a peristaltic pump through 10, 0.8, and 0.22-μm pore-size polycarbonate membranes (142 mm diameter, Millipore) to collect: large particle-attached (LPA, 10–20 μm), large free-living/small particle-attached (LFL/SPA, 0.8–10 μm), and free-living (FL, 0.22–0.8 μm) microbial fractions. To prevent filter clogging and minimize the retention of FL microorganisms, we replaced the membrane filter at each pore size (10, 0.8, and 0.22 μm) after processing approximately 5 L. All filters were immediately flash-frozen in liquid nitrogen and stored at -80°C until DNA extraction.

Seawater from the same three depth layers for inorganic nutrient analysis was filtered through 0.45-μm pre-combusted (450°C, 4 h) cellulose acetate membranes (47 mm diameter, Whatman) and immediately frozen at -20°C. The concentrations of nitrate (NO_3_^–^), nitrite (NO_2_^–^), dissolved inorganic phosphate (DIP), and dissolved silicate (DSi) were measured using standard colorimetric methods on a Technicon AA3 Auto-Analyzer (Blanc-Luebee, Germany) (Dai et al., 2008). The concentration of ammonium (NH_4_^+^) was determined by the indophenol blue spectrophotometric method (Pai et al., 2001). Additional environmental parameters, including temperature, salinity, depth, DO concentration, pH, turbidity and chlorophyll *a* concentration, were measured using a conductivity-temperature-depth (CTD; SBE 911plus) profiler equipped with multiparameter sensors. In this study, seawater with DO levels intermediate between hypoxic (≤ 2 mg L^–1^) and oxygen-saturated conditions (≥ 6 mg L^–1^) was operationally defined as “low-oxygen seawater” (Han et al., 2022).

### 2.2 Isotope-tracer measurements of nitrification rates

To determine ammonia (NH_3_) and nitrite (NO_2_^–^) oxidation rates, we conducted incubations spiked with ^15^NH_4_Cl and Na^15^NO_2_ tracers. For each depth and process, six 100 mL acid-washed glass serum bottles were purged with ≥ 2 volumes of sample water and then overflow-filled and sealed without headspace. Tracers were injected using gas-tight syringes to final concentrations of 500 nmol L^−1^ (≈10–20% of *in situ* NH_4_^+^) and 70 nmol L^−1^ (≈5–10% of *in situ* NO_2_^–^) for ^15^NH_4_Cl (ammonium oxidation) and Na^15^NO_2_ (nitrite oxidation), respectively (Raimbault and Garcia, 2008; Smith et al., 2014; Beman et al., 2021). For each sampled depth, water samples were incubated in temperature-controlled chambers set to the CTD-measured *in situ* temperature. Incubations were conducted for 0 (filtered immediately after tracer addition), 6, and 12 h, with two replicates per time point. After incubation, approximately 40 mL of seawater from each bottle was filtered through 0.22 μm polycarbonate membranes, and the filtrates were stored at −20°C until analysis.

The nitrogen isotope ratios δ^15^N-NO_*x*_^–^ were measured using the denitrifier method (Sigman et al., 2001; Casciotti et al., 2002). Briefly, ammonia oxidation products (NO_2_^–^ and NO_3_^–^) and nitrite oxidation products (NO_3_^–^) were converted to N_2_O by the denitrifying bacterium *Pseudomonas aureofaciens* (ATCC No.13985), and the δ^15^N values of N_2_O were then measured by Thermo Finnigan Gasbench–coupled stable isotope ratio mass spectrometer (GC-IRMS, Thermo Delta V Advantage). To quantify nitrite oxidation rate, samples were pretreated with sulfamic acid (≥ 99%, Sigma-Aldrich) for 12 h at room temperature (22–26°C) in the dark to remove NO_2_^–^ (Granger and Sigman, 2009), and subsequently neutralized with sodium hydroxide. The δ^15^N values of NO_*x*_^–^ were calibrated against three international isotopic references materials (USGS 34, IAEA-N3, and USGS 32) and a laboratory working standard, using a linear regression of measured versus assigned values (*R*^2^ > 0.999). Rates of ammonia oxidation in Equation 1 and nitrite oxidation in Equation 2 were calculated from the calibrated isotopic data using the following equation:


$$R_{\text{AO}}=\frac{d{[}^{15}{\mathrm{NO}}_{2}^{-}]}{d\,t}=\frac{{[}^{14}{\mathrm{NH}}_{4}^{+}]+{[}^{15}{\mathrm{NH}}_{4}^{+}]}{{[}^{15}{\mathrm{NH}}_{4}^{+}]}$$ (1)

$$R_{\text{NO}}=\frac{d{[}^{15}{\mathrm{NO}}_{3}^{-}]}{d\,t}=\frac{{[}^{14}{\mathrm{NO}}_{2}^{-}]+{[}^{15}{\mathrm{NO}}_{2}^{-}]}{{[}^{15}{\mathrm{NO}}_{2}^{-}]}$$ (2)

where *R*_AO_ and *R*_NO_ represent the ammonia oxidation and nitrite oxidation rate, respectively; *t* is the incubation time; [^15^NO_2_^–^] and [^15^NO_3_^–^] are the concentrates of ^15^N in NO_2_^–^ and NO_3_^–^ pools; and [^14^NH_4_^+^], [^14^NO_2_^–^], [^15^NH_4_^+^], and [^15^NO_2_^–^] are the concentrations of the natural abundance substrates and the artificial added stable isotopic tracers, respectively.

### 2.3 DNA extraction and metagenomic sequencing

DNA was extracted using a modified phenol-chloroform-isoamyl alcohol method (Massana et al., 1997), with concentrations quantified by a Qubit™ dsDNA HS Assay Kit (Invitrogen Qubit™, Thermo Fisher Scientific, MA, United States). Detailed information on DNA extraction is provided in Supplementary Table 1. Metagenomic libraries were prepared and sequenced on an Illumina Novaseq 6,000 platform (PE 2 × 150, Majorbio Bio-Pharm Technology Co., Ltd. in Shanghai, China). Raw sequences were quality-controlled using Fastp (Pearson and Lipman, 1988) to generate high-quality clean reads. *De novo* assembly was performed with MEGAHIT v1.2.9 (Li et al., 2016) followed by quality assessment using QUAST (Gurevich et al., 2013), retaining contigs ≥ 500 bp for downstream analysis. Open Reading Frames (ORF) prediction was conducted with Prodigal v.2.6.3 (Hyatt et al., 2010) in metagenomic mode (-p meta), excluding ORFs < 100 bp. A non-redundant gene catalog was generated using CD-HIT v4.8.1 (Fu et al., 2012) with identity (-c 0.9) and coverage (-aS 0.9) thresholds, selecting the longest sequence per cluster as representative. Clean reads from each sample were mapped to the non-redundant gene catalog using BWA v0.7.17 (Li and Durbin, 2010), with gene coverage calculated via CheckM v1.0.18 (Parks et al., 2015). For genes in the non-redundant gene catalog, relative abundance was quantified as reads per million [RPM = (mapped read counts/gene length)/sum of (mapped read counts/respective gene length)]. Because quantitative metagenomics with internal standards was not performed (Gifford et al., 2020), comparisons among size fractions reflect relative gene abundance patterns rather than absolute abundances. Nevertheless, to provide a more quantitative perspective, we conducted a DNA-yield-normalized comparison for key genes. This normalization was calculated as: gene abundance (RPM) × DNA yield per liter of seawater (μg L^–1^). Taxonomic and functional annotation was conducted through parallel analysis against multiple databases. DIAMOND v.2.1.7.161 (Buchfink et al., 2021) was employed with an *e*-value cutoff of 1 × 10^–5^ for searches against both the NCBI Non-Redundant Protein Sequence Database (NR) and the eggNOG database (Huerta-Cepas et al., 2016), while KofamScan v1.3.0 (Aramaki et al., 2020) was specifically used for Kyoto Encyclopedia of Genes and Genomes (KEGG) annotations (Kanehisa and Goto, 2000). In all cases, the top-scoring hits meeting database-specific criteria were retained, applying score thresholds for KEGG assignments and *e*-value cutoffs for NR and eggnog matches.

### 2.4 Metagenomic binning and annotation

Clean reads from all nine samples were co-assembled into contigs using MEGAHIT v1.2.9 (Li et al., 2016). Metagenome-assembled genomes (MAGs) were generated through binning with MetaWRAP v1.3.2 (Uritskiy et al., 2018) employing multiple algorithms (–metabat2 –maxbin2 –concoct). The resulting bins were consolidated using MetaWRAP’s bin refinement module, with medium-quality MAGs defined as those exhibiting ≥ 50% completeness and ≤ 10% contamination. All MAGs were dereplicated at 95% average nucleotide identity (ANI) using dRep v3.4.3 (Olm et al., 2017). Taxonomy of non-redundant MAGs was annotated with GTDB-TK v2.1.1 (Chaumeil et al., 2019) based on the GTDB r214 database (Parks et al., 2022). Gene prediction was performed using Prokka v1.11 (Seemann, 2014). For phylogenetic placement, domain-specific single-copy marker genes (120 for bacteria, 122 for archaea) were identified with HMMER v3.2.1 (Eddy, 2011), followed by sequence alignment through the gtdbtk align module and genome positioning on the reference tree using pplacer (Matsen et al., 2010).

### 2.5 Community reconstruction of AOA in Dongji Island’s coastal low-oxygen zone

The 843 AOA reference genomes were obtained from NCBI GenBank (Benson et al., 2013), JGI-IMG (Chen et al., 2022), and GOMC (Chen et al., 2024), then dereplicated at 97% average nucleotide identity (ANI) using dRep v3.4.3 (Olm et al., 2017). These dereplicated 342 genomes, combined with four MAGs recovered from this study, were used to construct a maximum-likelihood phylogenetic tree based on concatenated sequences of 53 single-copy core genes. Multiple sequence alignment was performed with GTDB-Tk (as described in section 2.4) (Chaumeil et al., 2019), followed by tree inference using IQ-TREE v2.2.0.3 (-m MFP -bb 1,000) (Nguyen et al., 2015). The phylogenetic tree was visualized using Chiplot (Xie et al., 2023). AOA genes were predicted using Prokka v1.14.6 (Seemann, 2014) and functionally annotated against the KEGG Orthology database (KOs) (Kanehisa and Goto, 2000) and NCycDB (Tu et al., 2019) as described above. Annotation results were manually curated and filtered for downstream analysis. For genes predicted from MAGs, relative abundance was calculated as reads per kilobase per million [RPKM = (mapped read counts/gene length)/total read counts]. Genome abundance was estimated as reads per kilobase of genome per gigabase of metagenome [RPKG = (mapped read counts/genome length)/metagenome size].

### 2.6 Statistical analysis

A two-way analysis of variance (ANOVA) was conducted in SigmaPlot v15.0 (Systat Software) to evaluate the combined effects of depth zone and microbial lifestyle on dominant phylum-level taxa. *Post-hoc* multiple comparisons were performed using the Holm-Šidák method (Zhao et al., 2021). For pairwise comparisons between independent groups, non-parametric Wilcoxon rank-sum tests were implemented in SPSS v19 (IBM Corp.).

## 3 Results and discussion

### 3.1 A hotspot for nitrification formed in deoxygenated water

**FIGURE 1:**
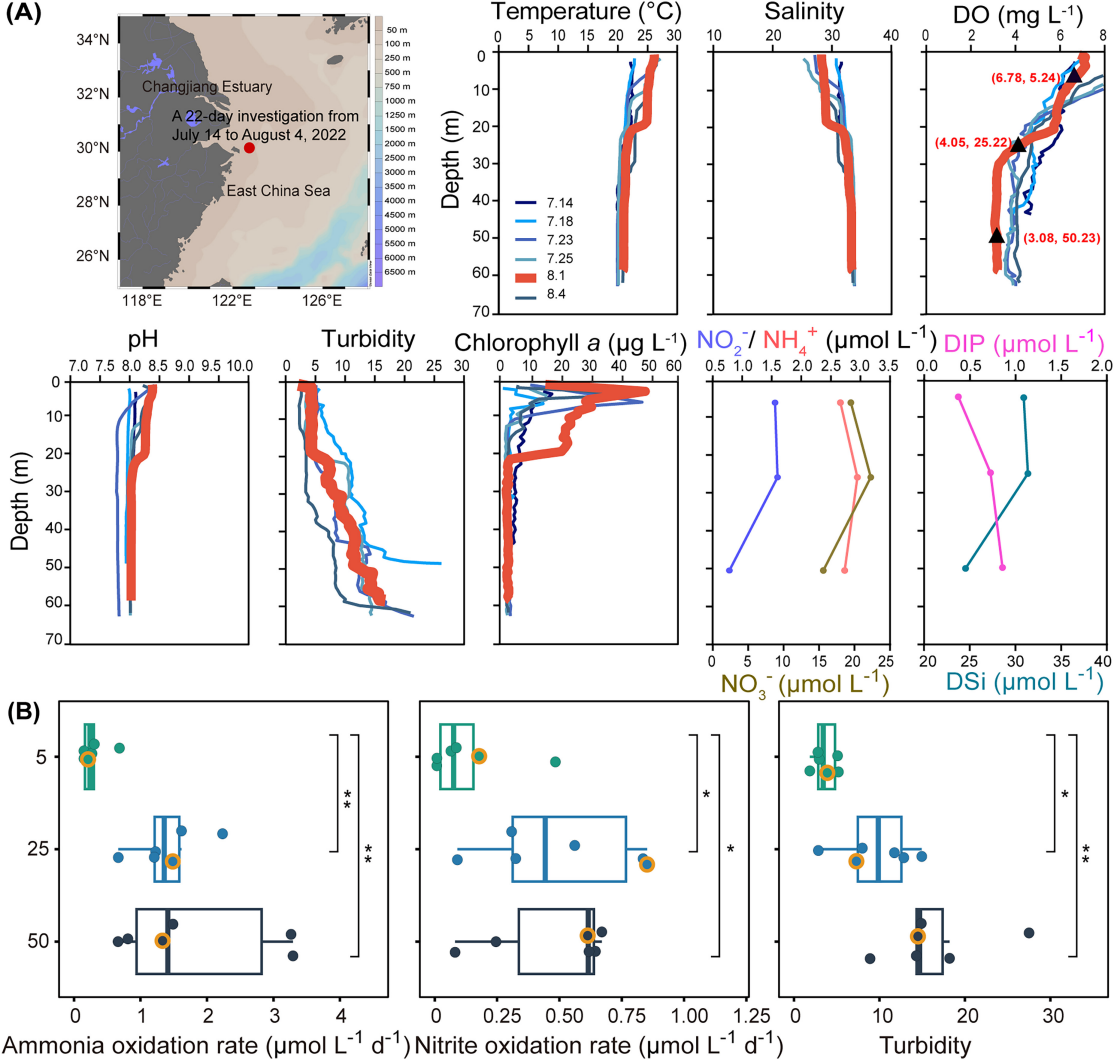
Study site and biogeochemical parameters. **(A)** Sampling site off Dongji Island (red dot) and depth-varying physicochemical parameters. Black triangles indicate the three typical biogeochemical layers of the water column: an oxic layer (5 m), an oxycline layer (25 m), and a low-oxygen layer (50 m). **(B)** Ammonia and nitrite oxidation rates and turbidity at three depths across six sampling occasions. Orange circles denote the day with the lowest dissolved oxygen (DO) concentration. DIP, dissolved inorganic phosphorus; DSi, dissolved silicate. *p < 0.05, **p < 0.01.

During the 22-day investigation, the study area surrounding Dongji Island (East China Sea) exhibited stable hydrological structure with pronounced vertical stratification (Figure 1A). This structure, characterized by distinct salinity gradients, can be attributed to the interplay between Changjiang river runoff and tidal mixing. When the most severe bottom-water oxygen depletion was observed on 1 August, the oxycline initiated at 20 m depth, coinciding with a sharp pycnocline where salinity increased abruptly from 30 to 32 and temperature dropped by 3°C (Figure 1A). Turbidity increased markedly below 20 m, indicating a strong tidal resuspension of bottom sediments (Turner and Millward, 2002). The pycnocline at 20 m acted as a physical barrier, trapping and concentrating suspended particles in the layer immediately beneath it. This accumulation of particles below the pycnocline severely attenuated photosynthetically active radiation. Phytoplankton biomass was therefore limited to the upper 20 m, as evidenced by the chlorophyll *a* maximum (49 μg L^–1^) at 2.5 m depth and its rapid attenuation to near-zero levels below the pycnocline (Figure 1A). The shallow chlorophyll maximum depth also aligned with the high turbidity in surface waters around Dongji Island, which was likely associated with particulate inputs from the Yangtze River (Sun et al., 2024). These terrestrial organic matters may enhance oxygen consumption in the surface layer, reducing the oxygen content available for downward transport. When coupled with water column stratification that restricts vertical mixing, this can lead to severely limited oxygen replenishment in deeper waters. Notably, the oxycline exhibited concurrent peaks in NH_4_^+^ and NO_3_^–^ concentrations, indicative of intense organic matter remineralization coinciding with oxygen depletion to ∼3 mg L^–1^ (Figure 1A). Together, terrestrial particle inputs and bottom sediment disturbance shaped a three-zone biogeochemical structure: oxygenated surface waters (0–20 m), a 10-m thick oxycline (20–30 m), and low-oxygen bottom waters (> 30 m), each driving distinct microbially mediated processes.

Over the near month-long investigation, ammonia oxidation rates ranged from 0.14 to 3.29 μmol L^–1^ d^–1^, while nitrite oxidation rates from 0.008 to 0.85 μmol L^–1^ d^–1^ (Figure 1B). These rates are comparable to those reported for other coastal and estuarine systems (Hsiao et al., 2014; Tang et al., 2022). Both ammonia and nitrite oxidation rates increased with declining DO, showing significantly higher values at the 25 and 50 m layers compared to the 5 m layer, respectively (*p* < 0.05–0.01). This vertical increase in nitrification rates coincided with a significant increase in turbidity at the 25 and 50 m layers relative to the 5 m layer (*p* < 0.05–0.01) (Figure 1A). These observations suggest that elevated nitrification in the more turbid, oxygen-depleted layers may be associated with particle-attached habitats. Previous studies have shown that suspended particles can substantially enhance ammonia oxidation in eutrophic coastal waters, likely through the *in situ* remineralization of particulate organic matter that supplies ammonium to fuel nitrification (Xia et al., 2009; Hsiao et al., 2014). Consistent with this possibility, size-fractionated tracer incubations have demonstrated that PA communities can account for the majority of total nitrification rates in some marginal seas (Zheng et al., 2017). However, without direct size-fractionated rate measurements, the partitioning of activity between PA and FL nitrifiers in our system awaits future quantification. Collectively, our findings suggest that nitrifiers in coastal particle-rich, oxygen-depleted waters may be predominantly associated with PA lifestyles.

### 3.2 AOA and NOB enrichment on particles in the low-oxygen zone

**FIGURE 2:**
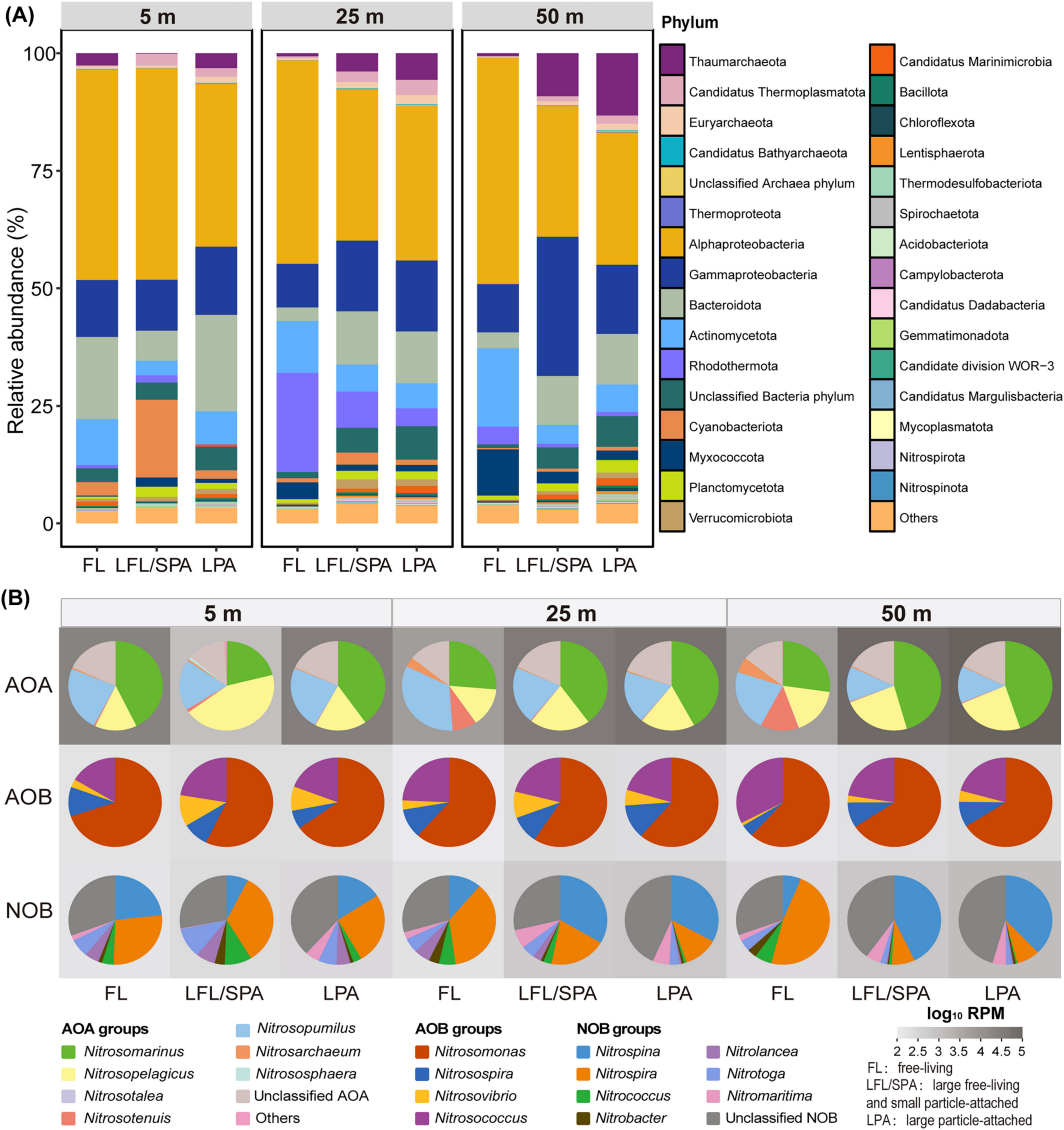
Microbial community composition across different depths and lifestyles. **(A)** Taxonomic profile at phylum level. **(B)** Ammonia-oxidizing archaea (AOA), ammonia-oxidizing bacteria (AOB), and nitrite-oxidizing bacteria (NOB) at genus level. The gray-scale heatmap in background represents the total abundance of each taxonomic group.

To evaluate the inferred relative enrichment in the PA size fraction of nitrifiers under these low-oxygen, particle-rich conditions, we selected seawater samples from the day of greatest oxygen depletion during our investigation for size-fractionated metagenomic sequencing. Comparing metagenomes across three depth zones (5, 25, and 50 m) and three lifestyles (FL, LFL/SPA and LPA), we found that microbial community compositions varied systematically along the oxygen gradient (i.e., depth gradient) (Figure 2A). Across all 187 detected prokaryotic phyla, bacteria dominated (83–99% relative abundance), while archaea constituted a minor fraction (e.g., ∼1% in the 50 m FL fraction) (Supplementary Table 2). However, archaeal phyla Thermoplasmatota and Euryarchaeota exhibited significant relative abundance differences between PA and FL lifestyles (ANOVA, *p* < 0.05; Supplementary Table 3), suggesting distinct niche partitioning. Pseudomonadota (mean 54.8%), Bacteroidota, Actinomycetota, and Rhodothermota comprised core heterotrophs, with Actinomycetota showing significant lifestyle-dependent variations (ANOVA, *p* < 0.05; Supplementary Table 3). Cyanobacteriota (3.1%) and Thaumarchaeota (4.3%) dominated autotrophic communities, while chemoautotrophic Nitrospirota and Nitrospinota were rare (< 0.1%).

Notably, the relative abundance of Thaumarchaeota increased as DO declined, exhibiting pronounced particle enrichment that was particularly evident in the low-oxygen zone (50 m) (Figure 2A). Marine AOA are typically submicron-sized (Könneke et al., 2005), so their detection on the > 0.8 μm fraction likely reflects attachment to particles/aggregates. Prior studies on deoxygenated waters in the Bohai Sea similarly reported a high relative abundance of both Thaumarchaeota genes and their corresponding transcripts under low oxygen (Han et al., 2022); moreover, pure-culture experiments have shown that marine AOA can physiologically sustain ammonia oxidation even at extremely low oxygen concentrations (Qin et al., 2017). However, most studies, including the Bohai Sea report, describe marine AOA as maintaining a predominantly FL lifestyle across both oxic and hypoxic conditions (Smith et al., 2013; Ganesh et al., 2014; Ijichi et al., 2019; Zhong et al., 2020; Han et al., 2022). This stands in contrast to our finding of a particle-associated (PA) enrichment here. Our results indicated that the high abundance of Thaumarchaeota was predominantly attributed to *Nitrosopelagicus* and *Nitrosomarinus* (Figure 2B), a pattern that contrasts with the typical dominance of *Nitrosopumilus* in coastal and estuarine systems (Smith et al., 2013; Reji et al., 2019; Rasmussen et al., 2022). This suggests habitat-specific niche partitioning among marine Thaumarchaeota clades, which is likely driven by their clade-specific adaptive strategies to varying oxygen gradients (Ulloa et al., 2012; Berg et al., 2015; Sollai et al., 2019).

Similarly, our metagenomic read recruitment against a comprehensive set of reference genomes (encompassing all available marine and typical non-marine AOA genomes from NCBI, GOMC, and JGI) (Supplementary Figure 1) revealed that *Nitrosomarinus*-like AOA and Water column group A (WCA; represented by *Nitrosopelagicus*)-like AOA were the dominant low-oxygen-adapted lineages at Dongji Island, both displaying a relative enrichment in the PA size fraction. This observation is notable because the WCA clade, which typically thrives in oxygen-rich shallow waters (Beman et al., 2008), appears here as a dominant lineage in a low-oxygen environment. In contrast (to the anomalous predominance of WCA), *Nitrosopumilus*-like AOA, a group typically associated with oxygenated waters, was itself rare and showed a preferential distribution in shallower, oxygenated depths in our study. Regarding the comparison between *Nitrosomarinus*-like and WCA-like AOA, the former showed a higher relative abundance, which aligns with its established dominance in marginal seas (Zou et al., 2019). This predominance may be attributed to its greater metabolic versatility, such as the possession of urease genes (Rasmussen et al., 2022). *Nitrosarchaeum*-like AOA were another abundant group in our low-oxygen zone, but they lacked a clear particle-colonization preference. Additionally, non-marine AOA genera were only rarely detected despite substantial terrestrial input here.

Ammonia-oxidizing bacteria (AOB) communities in the study area were primarily dominated by *Nitrosomonas*, followed by *Nitrosococcus* and *Nitrosospira* (Figure 2B). The relative contributions of AOA and AOB to ammonia oxidation vary across ecosystems and remain a subject of debate (Hatzenpichler, 2012). For instance, AOB can dominate in specific environments such as estuaries and hydrothermal vents (Mosier and Francis, 2008; Xu et al., 2014). In contrast, our study revealed a starkly different pattern in the low-oxygen zone off Dongji Island. Here, AOA exhibited a relative abundance two orders of magnitude higher than that of AOB, a disparity that was especially pronounced in the PA fractions (Figure 2B). This finding suggests that nitrification in this specific coastal low-oxygen habitat is primarily driven by AOA, which aligns with the prevailing pattern observed in the global open ocean (Molina et al., 2010; Luo et al., 2015; Peng et al., 2015). One potential explanation is that the scarcity of AOB in the low-oxygen zone reflects lower competitiveness for oxygen and/or ammonium relative to heterotrophs under organic-matter-rich conditions (Gieseke et al., 2001; Geets et al., 2006). Meanwhile, many marine AOA are characterized by higher affinities for oxygen and ammonia than canonical AOB, a physiological trait that may facilitate their persistence and activity under conditions of lower oxygen and substrate availability (Martens-Habbena et al., 2009).

*Nitrospina* and *Nitrospira* emerged as the dominant NOB in the waters surrounding Dongji Island (Figure 2B). While *Nitrospira* prevailed throughout oxic surface waters, oxygen depletion triggered distinct niche partitioning: *Nitrospira* remained dominant in the FL fraction. In contrast, *Nitrospina* dominated in the PA fraction, accounting for 40% of total NOB abundance and likely serving as the primary contributor to nitrite oxidation in the low-oxygen zone. Notably, although some *Nitrospina* cells can reach ∼6 μm in length (Spieck and Bock, 2015) and may therefore be partially retained in LFL/SPA fractions, *Nitrospina* remained consistently abundant in the LPA fraction, indicating that our overall distribution patterns are reliable. *Nitrospira* and *Nitrospina* were similarly observed as dominant NOB, exhibiting particle-enriched distributions in Pearl River estuary hypoxic waters (Hou et al., 2018). This shared PA preference by both AOA and NOB implies that particulate matter may serve as a key microhabitat for coupled ammonia oxidation and nitrite oxidation in the low-oxygen zone. This inference is reinforced by the fact that the concentration of PA DNA used for sequencing was consistently higher than that of FL DNA in the low-oxygen zone (Supplementary Table 1), which drove a consistently PA-enriched pattern in DNA-yield-normalized metagenomic abundances (Supplementary Figure 2). The probable reason for this PA-associated niche specialization is that the particulate organic matter surface provides localized NH_4_^+^ through ammonification, while tight spatial coupling between AOA and NOB can form “nitrifying aggregates” that enhance substrate transfer efficiency (Bottomley, 2006). Collectively, these results demonstrate that particulate matter serves as a critical ecological arena where DO-mediated selection and substrate microgradients jointly drive nitrifiers’ niche partitioning, revealing a previously underappreciated mechanism potentially sustaining nitrification in deoxygenating coastal seas.

### 3.3 Dominance of AOA functional gene in the low-oxygen zone

**FIGURE 3:**
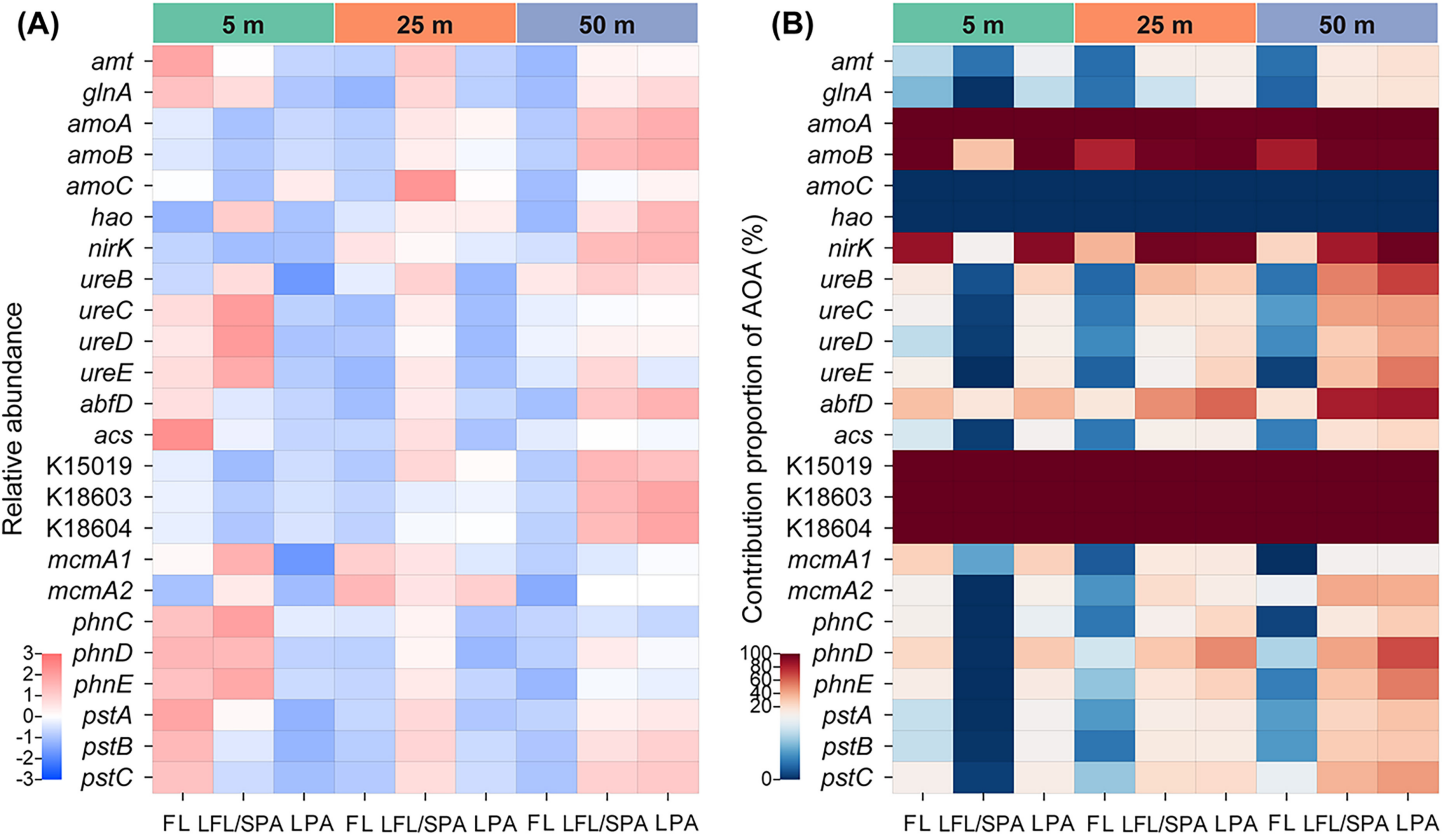
Distribution of key functional genes in the low-oxygen zone. **(A)** Total microbial community abundance (z-score normalized reads per million) and **(B)** AOA-specific contributions. *amt*, ammonium transporter; *glnA*, glutamine synthetase; *amoA*, ammonia monooxygenase subunit A; *amoB*, ammonia monooxygenase subunit B; *amoC*, ammonia monooxygenase subunit C; *hao*, hydroxylamine oxidoreductase; *nirK*, nitrite reductase (NO-forming); *ureB*, urease subunit beta; *ureC*, urease subunit alpha; *ureD*, urease accessory protein; *ureE*, urease accessory protein; *abfD*, 4-hydroxybutyryl-CoA dehydratase; *acs*, acetyl-CoA synthetase; K15019, 3-hydroxypropionyl-coenzyme A dehydratase; K18603, acetyl-CoA/propionyl-CoA carboxylase; K18604, acetyl-CoA/propionyl-CoA carboxylase; *mcmA1*, methylmalonyl-CoA mutase, N-terminal domain; *mcmA2*, methylmalonyl-CoA mutase, C-terminal domain; *phnC*, phosphonate transport system ATP-binding protein; *phnD*, phosphonate transport system substrate-binding protein; *phnE*, phosphonate transport system permease protein; *pstA*, phosphate transport system permease protein; *pstB*, phosphate transport system ATP-binding protein; *pstC*, phosphate transport system permease protein. FL, free-living; LFL/SPA, large free-living/small particle-attached; LPA, large particle-attached.

Building on the observed niche partitioning of nitrifiers in the low-oxygen zone, we further quantified the relative abundance of their dominant genes involved in key biogeochemical processes. The enrichment of ammonia transporter (*amt*) and assimilation (*glnA*) genes on particulate matter (0.8–20 μm) with depth, both in total community and specifically within AOA populations, reinforces their niche transition from FL to PA lifestyles, aligning with AOA’s overall particle preference (Figure 3). This metabolic transition may also influence phosphate utilization, as evidenced by the increasing relative abundance of *pstABC* genes in PA fractions with depth (Figure 3A). Phosphonate acquisition genes (*phnCDE*) did not exhibit this vertical pattern, likely due to the elevated DIP concentrations in the low-oxygen layers (Figure 1B). AOA contributions to both *pst* and *phn* genes increased with depth and showed particle-associated enrichment (Figure 3B). AOA’s dominant share of the urease genes (*ureBCDE*) identifies urea as a relevant ammonia source for these archaea in Dongji Island waters (Figure 3B). Their increasing prevalence with depth and strong association with larger particle fractions further raises the possibility that benthic sediment resuspension could serve as an important urea source to the water column. However, direct measurements of urea fluxes from resuspended sediments are needed to confirm this hypothesis. Notably, this AOA urea utilization profile closely corresponds to *Nitrosomarinus*’ strong preference for low oxygen, particle-associated niches, suggesting this genus likely dominates benthic urea-fueled ammonia oxidation in the region. The significant PA enrichment of ammonium monooxygenase genes (*amoA*, *amoB*, *amoC*) and nitrite reductase gene (*nirK*) suggests particulate matter serves as genetic hotspots for nitrogen cycling, particularly in bottom waters (Figure 3A). Nearly all detected *amoA*, *amoB*, and *nirK* genes originated from AOA, strongly suggesting their dominant role in driving ammonia oxidation in the low-oxygen zone (Figure 3B). Similarly, genes for the PA 3HP/4HB carbon fixation pathway detected in the low-oxygen environment predominantly belonged to AOA. Of course, strong tidal resuspension may also introduce dead or inactive cells onto particles in the bottom layer. Therefore, part of the observed gene enrichment could originate from those non-viable sources. However, even accounting for this potential contribution of dead or inactive biomass, the elevated nitrification rates observed in the bottom layer could still be supported by the metabolic potential indicated by the PA gene enrichment. The same trend persists after normalizing key metabolic gene abundances by DNA yield (Supplementary Figure 3), providing an additional line of evidence supporting the conclusion above. Overall, the particle-associated enrichment of key metabolic genes supports the view that AOA are central drivers of coupled nitrogen cycling and carbon fixation in the low-oxygen zone, with marine particles acting as “microreactors” that may facilitate these biogeochemical synergies.

### 3.4 Niche partitioning reshapes nitrogen cycling

**FIGURE 4:**
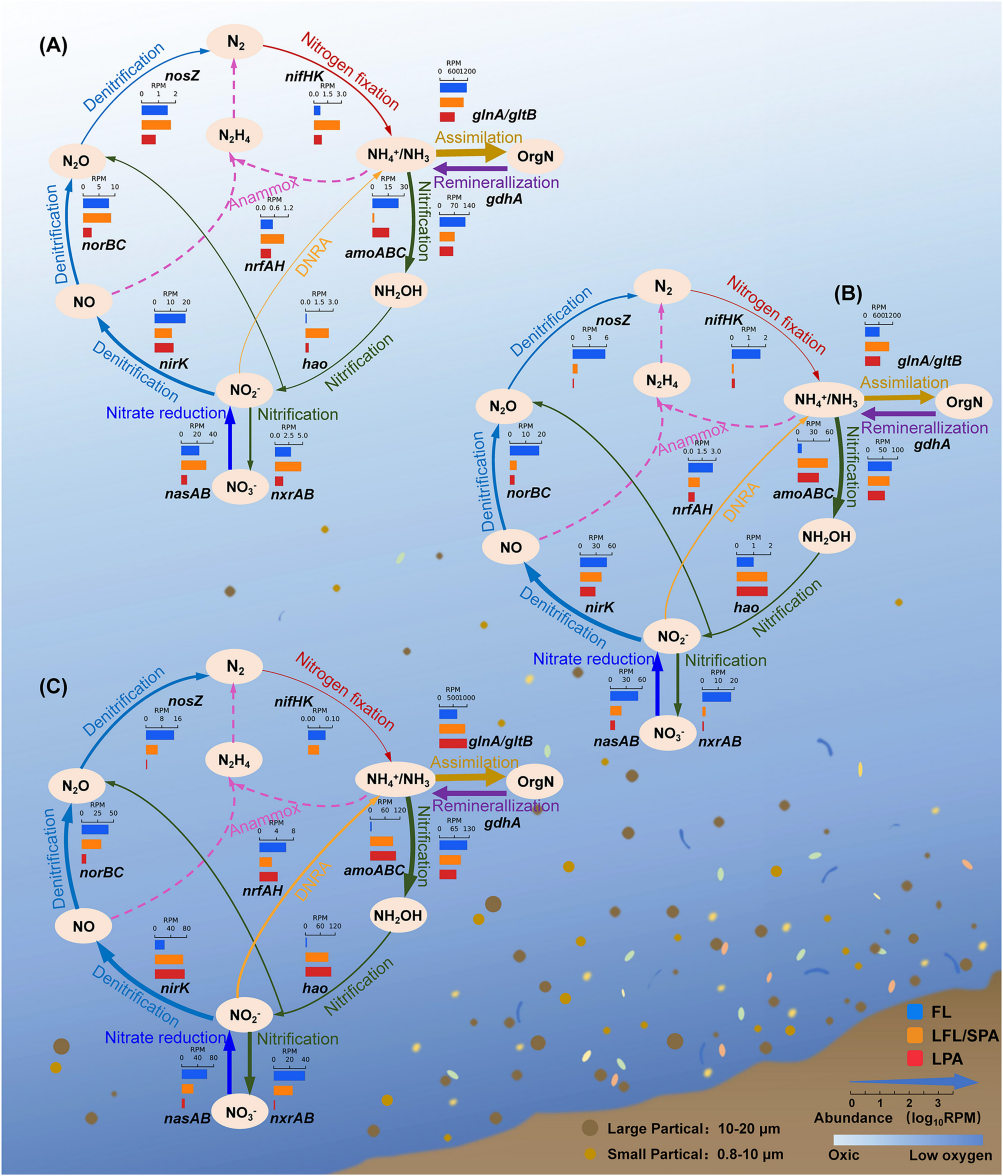
Relative abundance of microbial nitrogen cycling genes near Dongji Island. **(A)** 5 m; **(B)** 25 m; **(C)** 50 m. The thickness of the arrows corresponds to gene relative abundance levels (measured in reads per million, RPM). The bar charts represent the relative abundances of genes in three size fractions: free-living (FL), large FL/small particle-associated (LFL/SPA), and large particle-associated (LPA). Dotted arrows indicate the absence of genes required for the processes.

The increasing relative enrichment of AOA for PA lifestyles with depth may generate cascading effects throughout the nitrogen cycle. To assess these effects, we analyzed the relative abundance and DNA-yield-normalized abundance distribution of nitrogen-cycle functional genes along the oxygen (depth) gradient and across particle size fractions (Figure 4 and Supplementary Figure 4). We observed a systematic shift in *amoABC* gene dominance from FL to PA communities with depth and declining DO, while hydroxylamine oxidoreductase (*hao*) maintains a persistent PA preference throughout the DO gradient. This highlights the critical role of particles as microhabitats associated with ammonia oxidation in the low-oxygen zone (Figures 4B,C). In contrast to the strictly PA distribution of ammonia oxidation genes, nitrite oxidation genes (*nxrA*/*nxrB*) exhibit a distinct distribution pattern, indicating that FL communities may still substantially contribute to nitrite oxidation, despite the general PA preference of NOB. Notably, over 70% of the *nxr* gene abundance was contributed by Gammaproteobacteria and Alphaproteobacteria, rather than by classic NOB. An additional possibility is the presence of novel *nxr* genes that are not yet annotated in this unique environment. However, when *nxr* gene abundance was normalized to DNA yield, the dominant *nxr* genes were still primarily associated with the PA fraction (Supplementary Figure 4).

The denitrification marker *nirK* showed predominant association with PA fractions, accounting for 86% of total *nirK* at 50 m depth (Figure 4C), with significantly higher relative abundance than *nirS* (Supplementary Figure 5). This *nirK* dominance aligns with its prevalence in low-oxygen zones of the Pearl River Estuary, the Jiulong River, and the Bohai Sea (Zou et al., 2019; Zou et al., 2020; Han et al., 2022). These cross-system similarities suggest that *nirK*-type denitrifiers may exhibit broader habitat adaptability compared to *nirS*-type communities (Jones and Hallin, 2010; Helen et al., 2016). The observed co-enrichment of *nirK* and *amoA* (along with *amoB*) on particles in the low-oxygen zone suggests a potential coupling between AOA-driven nitrite production and its consumption in these microenvironments. This potential coupling could be mediated by shared substrate gradients (e.g., NH_4_^+^→NO_2_^–^→NO) as proposed in previous research (Zhang et al., 2014). Genes encoding nitric oxide reductase (*norBC*) and nitrous oxide reductase (*nosZ*) were 4–5-fold more abundant in the hypoxic bottom layer than in surface waters (Figure 4 and Supplementary Figure 5), further indicating enhanced denitrification potential in this low-oxygen zone. Previous research has demonstrated that particle-associated microenvironments can expand denitrifiers’ ecological niches, establishing them as key nitrous oxide (N_2_O) producers even in oxygenated coastal waters (Wan et al., 2023b). Our study revealed a distinct partitioning pattern of denitrification genes in the low-oxygen zone: *nirK* showed preferential enrichment in particle-associated habitats, while *norBC* and *nosZ* were enriched in the FL fraction (Figure 4). This decoupling suggests that NO produced in particles may rapidly diffuse into the surrounding water column, potentially influencing FL communities. In addition, *nrfAH* genes involved in dissimilatory nitrate reduction to ammonium (DNRA) were enriched in the low-oxygen zone. This nitrogen retention (resulting in ammonia supply) could potentially enhance local biological production (Michiels et al., 2017). No anaerobic ammonium oxidation (anammox) genes were detected in the study environment. These inferences are further supported by DNA-yield-normalized gene abundances, which confirm the predominant enrichment of the discussed nitrogen-cycling genes in the PA fraction (Supplementary Figure 4). Finally, we note that while the DNA-yield-normalized abundances provide a more quantitative comparison than relative proportions alone, they still represent gene enrichment patterns rather than absolute quantities or direct process rates. Moving from relative patterns to robust quantitative comparisons across size fractions will require absolute quantification (e.g., via a synthetic spike-in standard for 16S amplicon sequencing or quantitative metagenomics) (Gifford et al., 2020; Cram et al., 2024; Huanca-Valenzuela et al., 2025), which is a key objective for future work.

### 3.5 Clade-divergent strategies in AOA niche partitioning

To elucidate the unique microbial adaptations in this low-oxygen zone, we reconstructed 253 medium-quality non-redundant metagenome-assembled genomes (MAGs; completeness ≥ 50%, contamination ≤ 10%), including 64 high-quality MAGs (completeness ≥ 90%, contamination ≤ 5%). Taxonomic classification assigned 233 bacterial and 20 archaeal MAGs, spanning 17 bacterial and 3 archaeal phyla (Supplementary Figures 6A,C and Supplementary Table 4). Proteobacteria (*n* = 95) dominated the bacterial community, comprising 54 Alphaproteobacteria and 41 Gammaproteobacteria. Other prevalent bacterial phyla were Bacteroidota (*n* = 49), Actinobacteriota (*n* = 24), and Planctomycetota (*n* = 18). Among archaea, Thermoplasmatota (*n* = 15) was most abundant, followed by Thermoproteota (*n* = 4) and Asgardarchaeota (*n* = 1). Notably, 72% of bacterial (168/233) and 70% of archaeal (14/20) MAGs exhibited genomic novelty (average nucleotide identity, ANI < 95% to reference genomes), including 9 novel families and 37 novel genera (Supplementary Figure 6B). This highlights the substantial taxonomic novelty of microbial communities in the low-oxygen environment.

**FIGURE 5:**
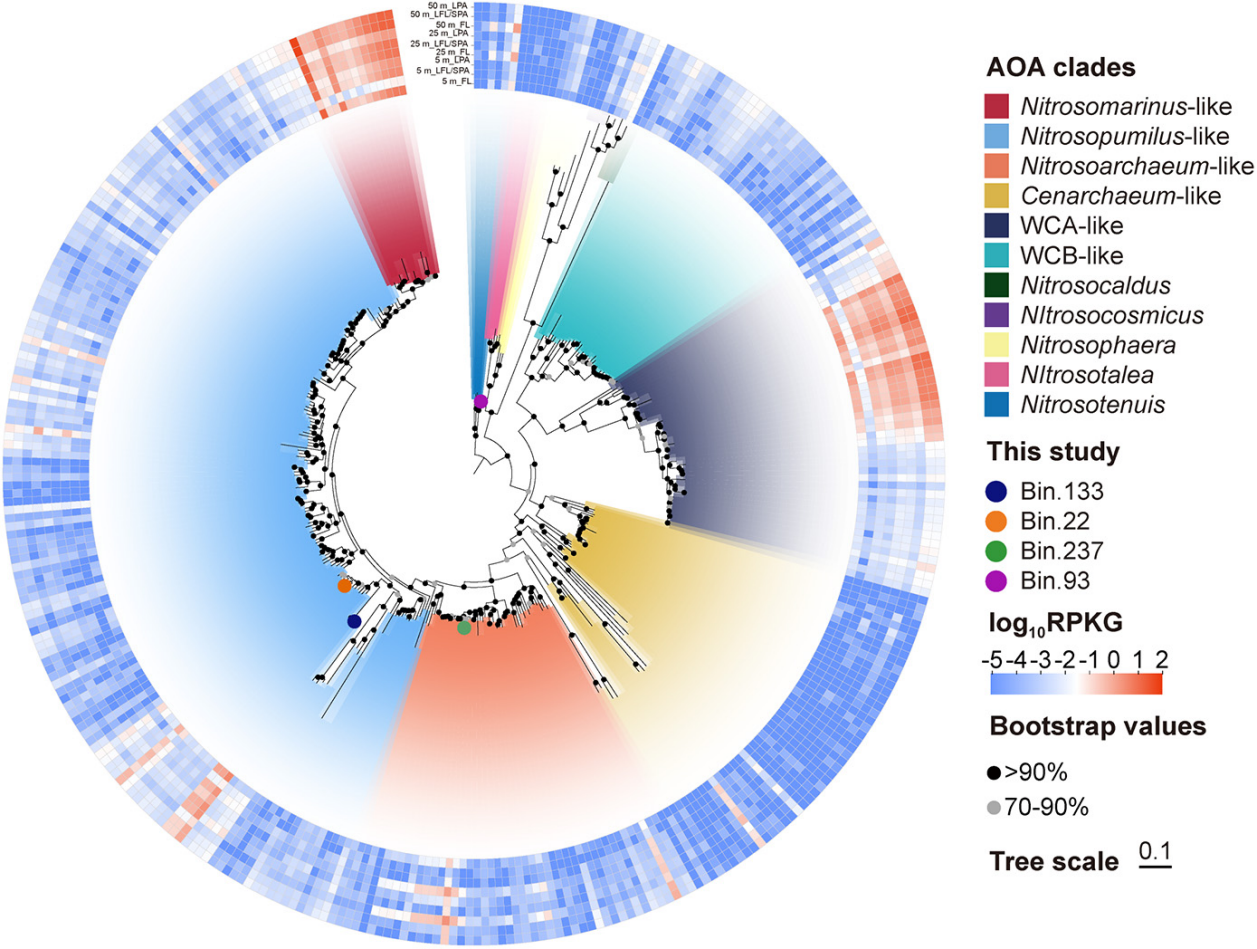
Phylogenetic reconstruction and relative abundance patterns across 346 archaeal ammonia-oxidizing (AOA) genomes. AOA genomes obtained from this study are highlighted with solid circles on branches. The inner ring’s color scheme denotes AOA clades; the outer heatmap shows their relative abundance across nine samples (expressed as RPKG: reads recruited per kilobase of metagenome-assembled genomes per gigabase of metagenome). Branch support values were calculated from 1,000 bootstrap replicates. The scale bar indicates 10% estimated sequence divergence.

We recovered four medium-quality AOA MAGs from Nitrososphaeria, representing three distinct genera. Bin.237 clustered with *Nitrosarchaeum*, a lineage initially described in freshwater ecosystems (Tian et al., 2020) but now recognized as cosmopolitan in open oceans (Qin et al., 2020). Bin.93 aligned with the soil-derived *Nitrosotenuis* (Jung et al., 2014), while bin.133 and bin.22 matched the marine genus *Nitrosopumilus.* To enable a comprehensive profiling of the *in situ* AOA community through metagenomic read recruitment, we supplemented our limited number of AOA MAGs by downloading all accessible AOA genomes from global marine and select freshwater environments. The four novel AOA MAGs were integrated with 342 publicly available marine AOA genomes and representative non-marine AOA references to construct a comprehensive phylogenetic tree. According to the established phylogenomic framework (Qin et al., 2020), the tree comprised 11 distinct AOA clades (Figure 5). Metagenomic read recruitment to these AOA genomes revealed that *Nitrosomarinus*-like AOA and WCA-like AOA dominated the low-oxygen waters of Dongji Island (Figure 5 and Supplementary Figure 1). This contrasts with previous findings where *Nitrosopumilus* prevailed in intertidal sediments of the Zhoushan Islands (Hu et al., 2019). This distinct community composition at Dongji Island implies specific adaptations to the low-oxygen conditions at our study site.

**FIGURE 6:**
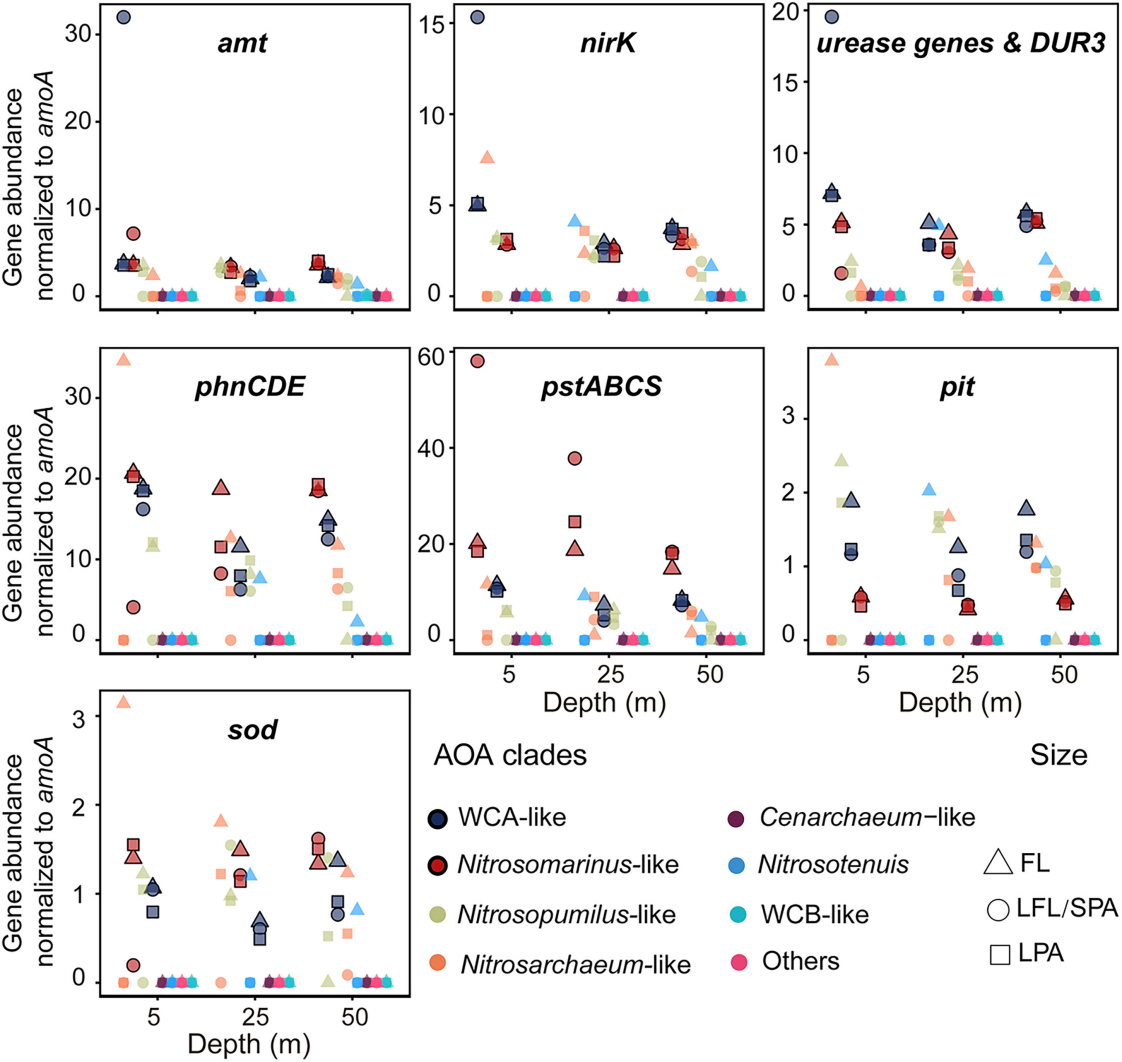
Relative abundance of key functional genes, normalized to their corresponding *amoA* gene copy number, across AOA clades. Colors correspond to AOA phylogenetic clades. Symbol shapes represent distinct lifestyles: free-living (FL), small particle-associated (SPA), and large particle-associated (LPA) fractions. “Others” include *Nitrosocaldus*, *Nitrosocosmicus*, *Nitrosotalea*, and *Nitrososphaera*. *amt*, ammonium transporter; *nirK*, nitrite reductase (NO-forming); *ureABC*, urease; URE, urease; DUR3, urea-proton symporter; *sod*, superoxide dismutase; *phnCDE*, phosphonate transporters; *pstABCS*, high-affinity phosphate transporter; *pit*, low-affinity phosphate transporter.

To assess metabolic specialization across these 11 clades, we quantified the relative abundance of key functional genes, normalized to their corresponding *amoA* gene copies (RPKG) (Figure 6). We focused on comparing three key AOA groups in this study region: *Nitrosomarinus*-like, WCA-like, and *Nitrosopumilus*-like AOA. Given their broadly comparable MAG completeness and contamination levels (as indicated by CheckM), we consider it unlikely that the observed relative abundance differences stem from systematic genome-quality bias. Our results indicated that *Nitrosomarinus*-like AOA exhibited the highest *amt*-to-*amoA* ratio (mean ± SD = 3.91 ± 1.28, *n* = 9 across the water column), which was significantly higher than those of *Nitrosopumilus*-like AOA (2.15 ± 1.42; Wilcoxon rank-sum test, *p* < 0.05) and WCA-like AOA (2.54 ± 0.71, *p* < 0.01, excluding an outlier). This enhanced ammonia acquisition capacity likely underpins *Nitrosomarinus*-like AOA’s ecological success across the water column. Additionally, *Nitrosomarinus*-like AOA and WCA-like AOA exhibited significantly higher *nirK*-to-*amoA* ratios than other clades in the low-oxygen bottom layer (*p* < 0.01), suggesting enhanced nitrite detoxification (Walker et al., 2010) that promotes competitiveness at the cost of potentially higher nitric oxide emissions under hypoxia (Löscher et al., 2012). Many marine Thaumarchaeota strains possess urease genes (*ure*) for urea utilization, enabling urea-dependent growth (Alonso-Sáez et al., 2012; Qin et al., 2014). Notably, both *Nitrosomarinus*-like AOA and WCA-like AOA exhibited significantly higher *ure* gene copy number than other clades (*p* < 0.01), reinforcing urea metabolism as a key niche-defining trait in these lineages. Consistent with the known requirement of AOA for oxidative stress mitigation even in hypoxic waters (Han et al., 2022), the prevalent clades at our study site retained superoxide dismutase genes (*sod*). However, WCA-like AOA’s significantly lower *sod*-to-*amoA* ratio (*p* < 0.05), particularly in PA fractions, suggests a potentially greater reliance on heterotrophic microbial partners for reactive oxygen species (ROS) detoxification (Bayer et al., 2019), an adaptation likely enabled by the metabolically cooperative microbial consortia characteristic of particle microenvironments.

Phosphorus acquisition strategies diverged markedly between the dominant archaeal clades in Dongji Island’s low-oxygen waters. *Nitrosomarinus*-like AOA maintained equally high gene potential for both high-affinity phosphate transporters (*pstABCS*) and phosphonate uptake systems (*phnCDE*). WCA-like AOA exhibited a stronger preference for the low-affinity Pit phosphate transporter system than *Nitrosomarinus*-like AOA (*p* < 0.01), potentially due to Pit’s energy-saving advantage over high-affinity Pst systems (Gebhard et al., 2009). Concurrently, WCA-like AOA maintained *phnCDE* genes for organophosphate utilization. Both *Nitrosomarinus*-like AOA and WCA-like AOA showed high genetic potential for dual inorganic/organic phosphorus uptake, an adaptation likely reflecting their co-occurrence with phytoplankton in the shallow waters where organophosphates are relatively abundant. In contrast, while *Nitrosopumilus*-like AOA displayed similar Pit preference to WCA-like AOA, its consistently lower *phnCDE* copy numbers (compared to both *Nitrosomarinus* and WCA; *p* < 0.01) may explain its competitive limitation in the low-oxygen niches. Consistent with the ambient low-phosphate concentration (< 1 μmol L^–1^; Figure 1A) at the study site, the dominant AOA populations maintained high genetic potential for at least one high-affinity transporter system (Pst or Phn) to facilitate efficient phosphorus acquisition in this region. These functional profiles reveal how *Nitrosomarinus*-like AOA and WCA-like AOA employ distinct strategies to adapt to particle-associated microniches, providing critical insights into AOA’s adaptive metabolic mechanisms under marine deoxygenation.

## 4 Conclusion

Our integrated dataset of ^15^N-tracer nitrification rates, metagenomics, and genome-resolved analysis indicates that elevated two-step nitrification rates with decreasing oxygen concentration were observed alongside distinct microbial community structures and metabolic partitioning between FL and PA fractions along the oxygen gradient in the Dongji Island marine ecosystem. The results reveal that marine deoxygenation drives nitrifier niche partitioning, resulting in the enrichment of PA nitrifiers in the low-oxygen zone and a strictly PA distribution of ammonia oxidation genes. PA AOA communities were dominated by the *Nitrosomarinus* and WCA clades, whose enriched urease genes suggest potential utilization of urea as an auxiliary nitrogen source. Clade-specific analyses demonstrated that *Nitrosomarinus*-like AOA and WCA-like AOA adapt to PA niches in low-oxygen environments through distinct metabolic strategies. Specifically, *Nitrosomarinus*-like AOA contained more gene copies for high-affinity ammonia transporters (*amt*) and phosphate transporters (*pst*) than WCA-like AOA, whereas WCA-like AOA retained more low-affinity phosphate transporters (*pit*) than *Nitrosomarinus*-like AOA. Notably, particle-associated AOA may directly couple nitrite production and consumption through co-enriched ammonium monooxygenase (*amoA*) and nitrite reductase genes (*nirK*). The resulting NO production could facilitate downstream denitrification processes, contributing to nitrogen loss. These findings reveal how AOA adaptations to particle-associated microenvironments influence nitrogen cycling and coupled biogeochemical responses under marine deoxygenation.

## Data Availability

All data are available in the main text or the Supplementary materials. Raw sequences generated in this study are deposited in the Sequence Read Archive under BioProject ID PRJNA1281586.
