# Supplementary material for: Niche adaptation of particle-associated ammonia-oxidizing archaea sustains nitrification under marine deoxygenation
Source: Front Microbiol. 2026 Mar 12;17:1773718. doi: 10.3389/fmicb.2026.1773718 (PMC13018155; doi:10.3389/fmicb.2026.1773718)
Supplement: Supplementary file 1 [file Data_Sheet_1.docx]

Supplementary Material


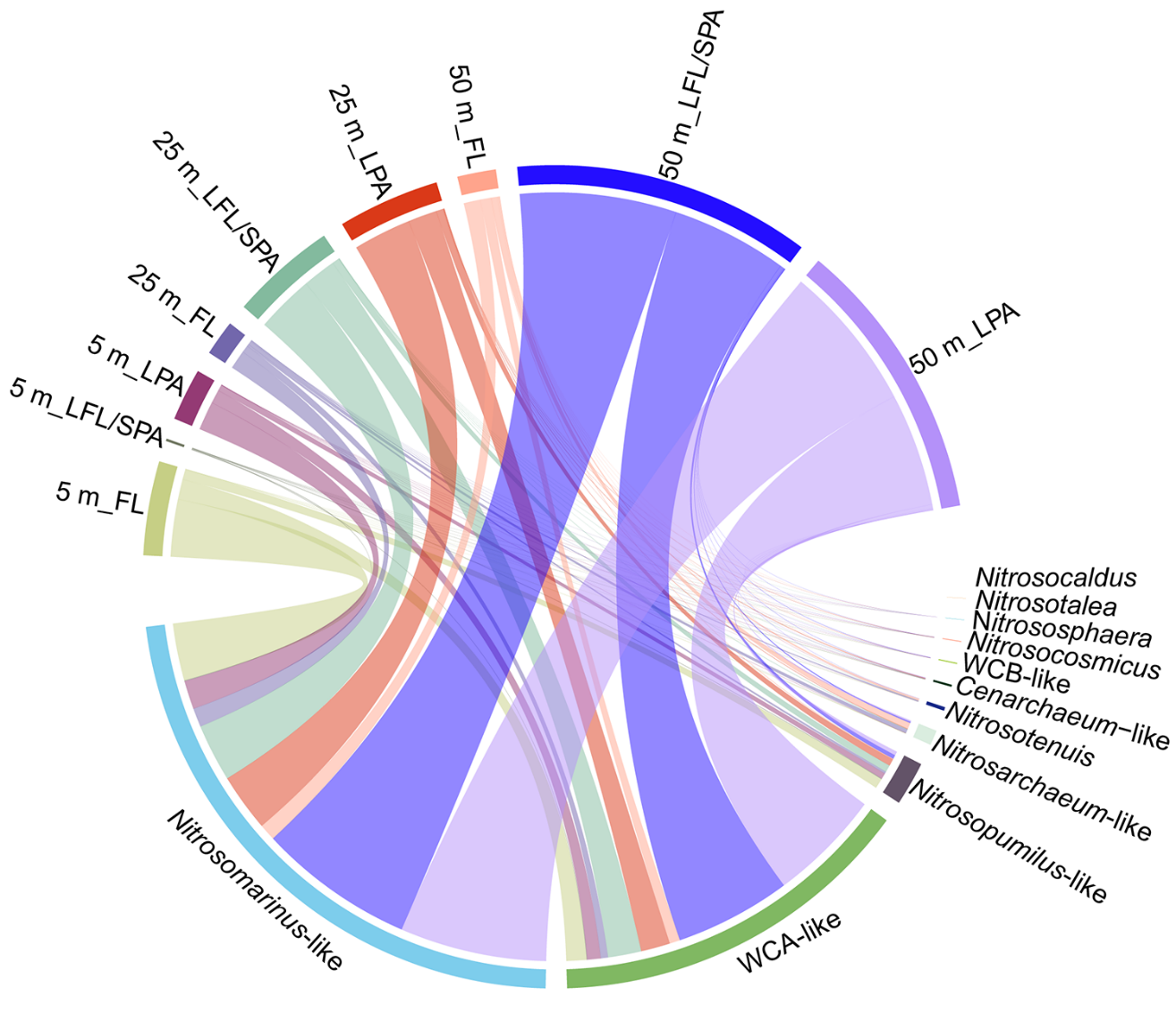


**Supplementary Figure 1.** Distribution of 346 AOA genomes (marine and typical non-marine) across different depths and lifestyles, grouped into 11 phylogenetically distinct clades. FL, free-living; LFL/SPA, large free-living/small particle-attached; LPA, large particle-attached.


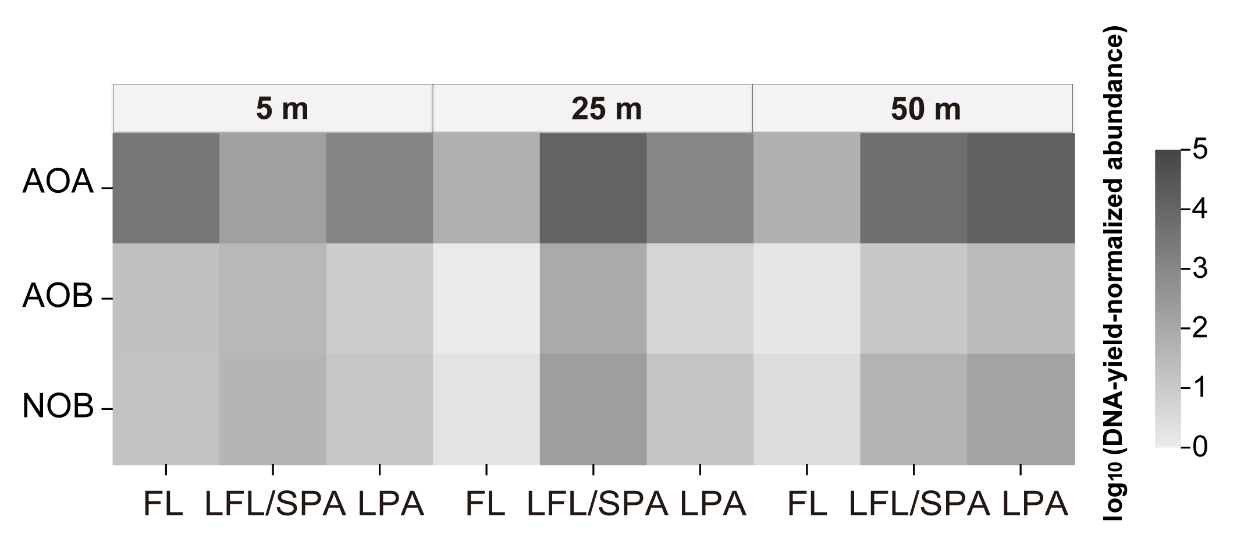


**Supplementary Figure 2.** DNA-yield-normalized genus-level total abundances of ammonia-oxidizing archaea (AOA), ammonia-oxidizing bacteria (AOB), and nitrite-oxidizing bacteria (NOB) across depths and size fractions. FL, free-living; LFL/SPA, large free-living/small particle-attached; LPA, large particle-attached.


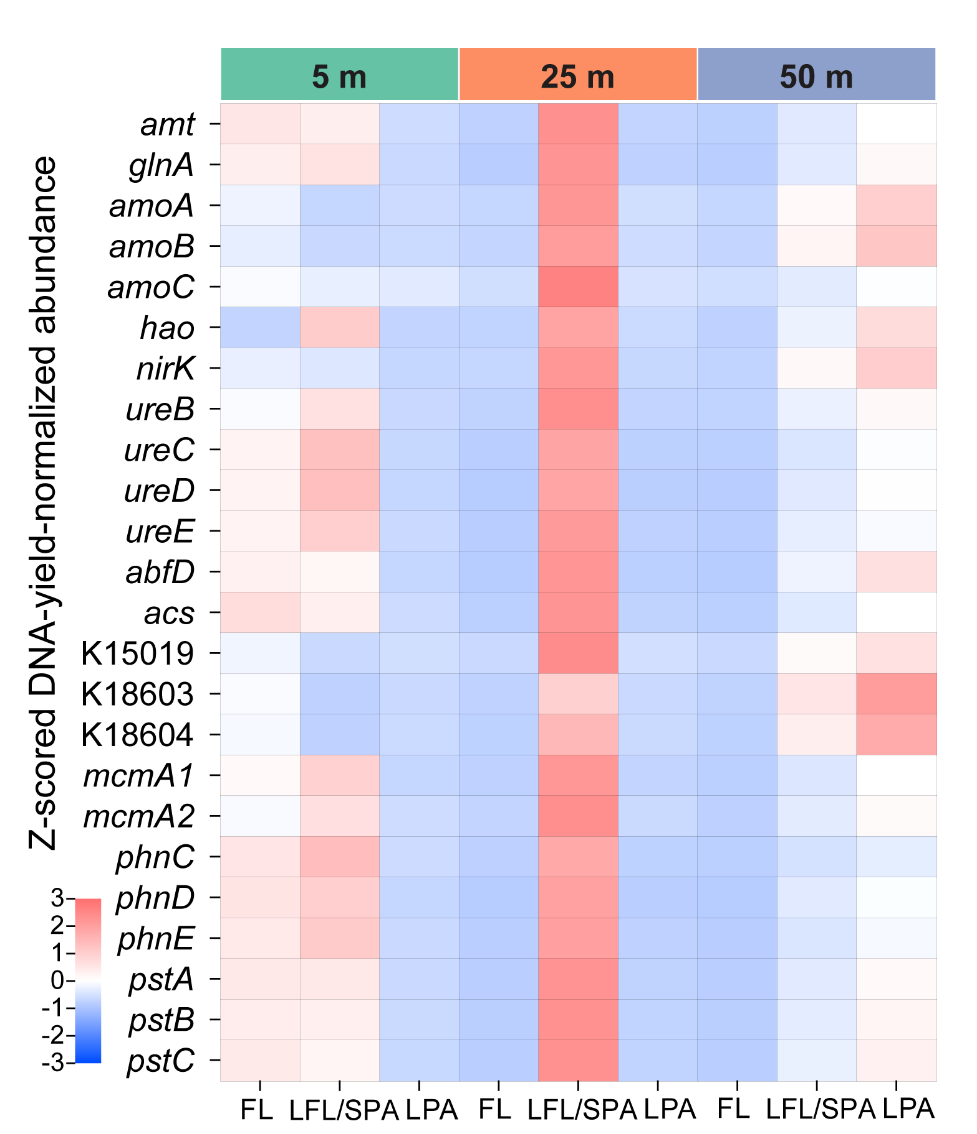


**Supplementary Figure 3.** Distribution of key functional genes in the low-oxygen zone based on Z-scored DNA-yield-normalized reads per million of the total microbial community. *amt*, ammonium transporter; *glnA*, glutamine synthetase; *amoA*, ammonia monooxygenase subunit A; *amoB*, ammonia monooxygenase subunit B; *amoC*, ammonia monooxygenase subunit C; *hao*, hydroxylamine oxidoreductase; *nirK*, nitrite reductase (NO-forming); *ureB*, urease subunit beta; *ureC*, urease subunit alpha; *ureD*, urease accessory protein; *ureE*, urease accessory protein; *abfD*, 4-hydroxybutyryl-CoA dehydratase; *acs*, acetyl-CoA synthetase; K15019, 3-hydroxypropionyl-coenzyme A dehydratase; K18603, acetyl-CoA/propionyl-CoA carboxylase; K18604, acetyl-CoA/propionyl-CoA carboxylase; *mcmA1*, methylmalonyl-CoA mutase, N-terminal domain; *mcmA2*, methylmalonyl-CoA mutase, C-terminal domain; *phnC*, phosphonate transport system ATP-binding protein; *phnD*, phosphonate transport system substrate-binding protein; *phnE*, phosphonate transport system permease protein; *pstA*, phosphate transport system permease protein; *pstB*, phosphate transport system ATP-binding protein; *pstC*, phosphate transport system permease protein. FL, free-living; LFL/SPA, large free-living/small particle-attached; LPA, large particle-attached.


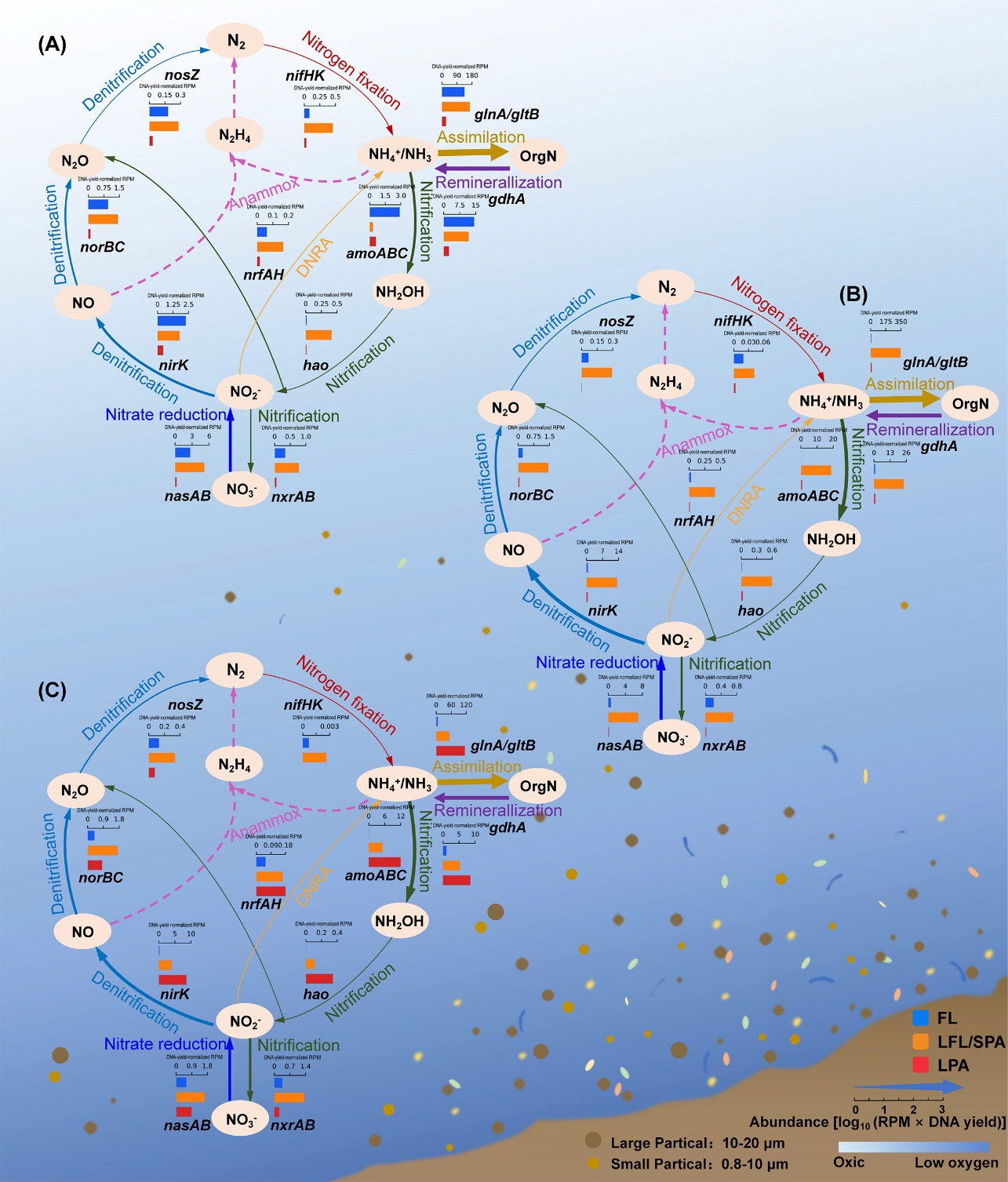


**Supplementary Figure 4.** DNA-yield-normalized gene abundance of microbial nitrogen-cycling genes near Dongji Island. (A) 5 m; (B) 25 m; (C) 50 m. The thickness of the arrows corresponds to DNA-yield-normalized gene abundance levels [measured in reads per million (RPM) × DNA yield per liter of seawater (µg L^-1^)]. The bar charts represent the relative abundances of genes in three size fractions: free-living (FL), large FL/small particle-associated (LFL/SPA), and large particle-associated (LPA). Dotted arrows indicate the absence of genes required for the processes.


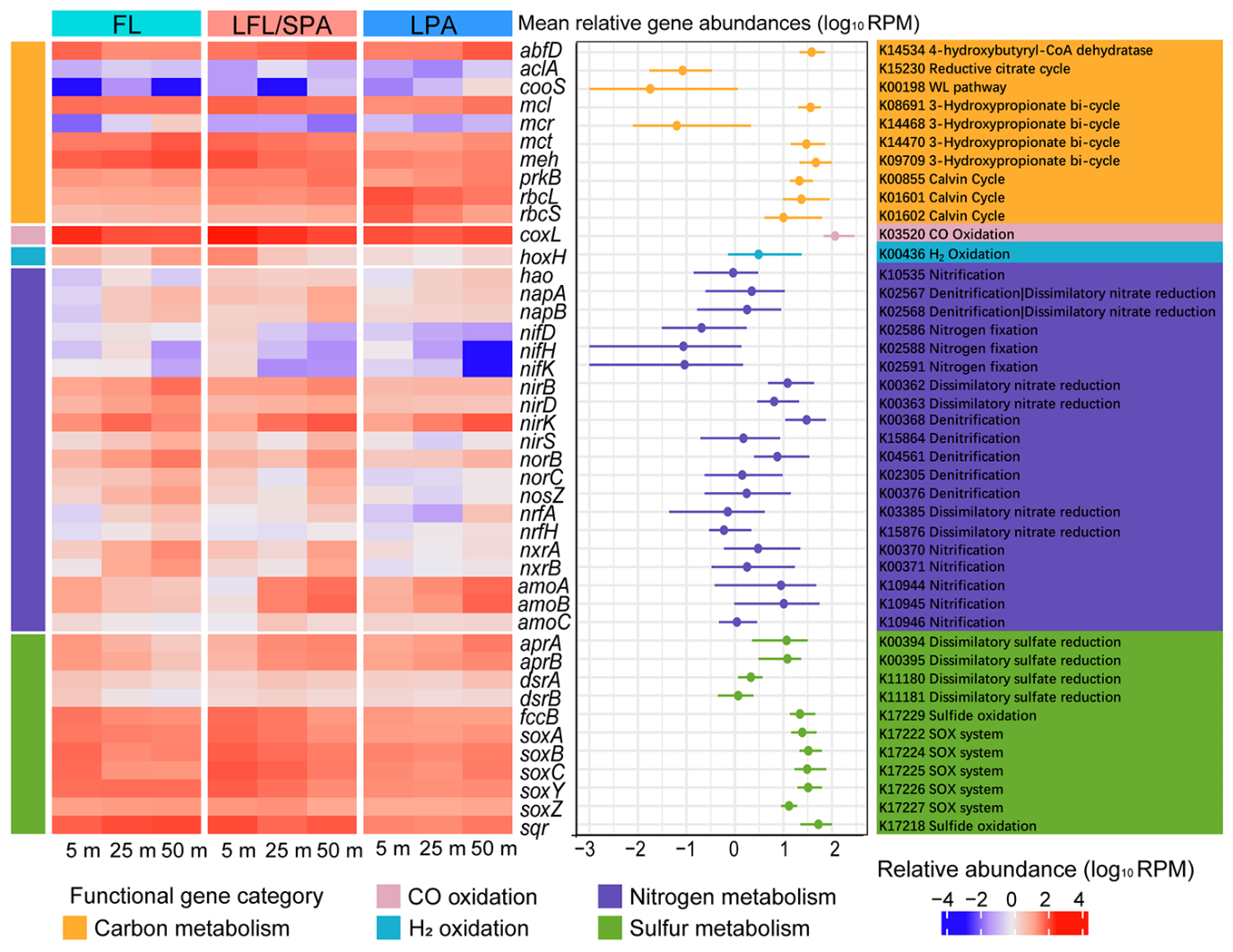


**Supplementary Figure 5.** Heatmap showing the relative abundance of 43 marker genes linked to key metabolic pathways: carbon, carbon monoxide (CO), hydrogen (H_2_), nitrogen, and sulfur metabolism. The central panel shows the average gene abundance across all samples. FL, free-living; LFL/SPA, large free-living/small particle-attached; LPA, large particle-attached.


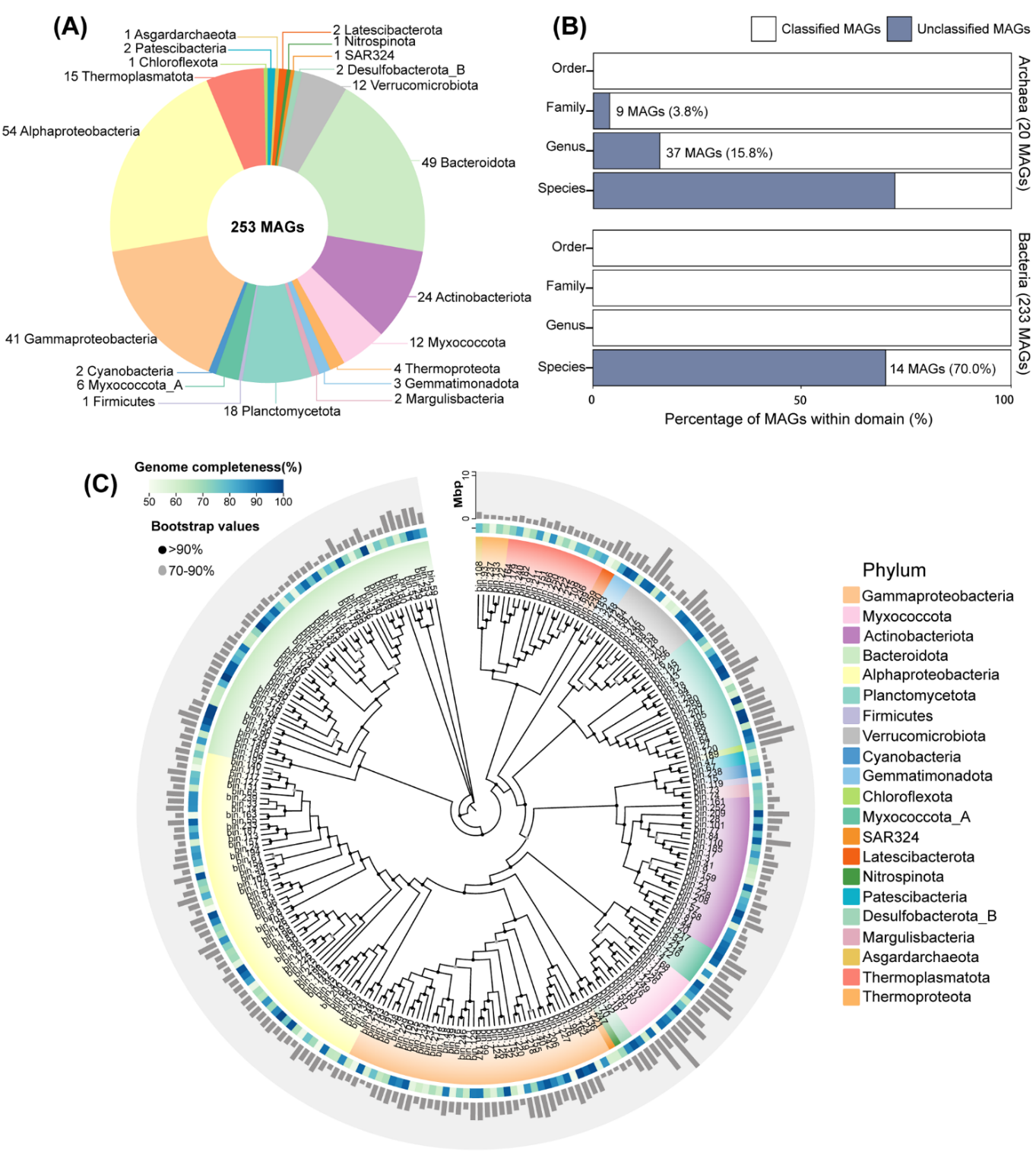


**Supplementary Figure 6.** Taxonomic and phylogenetic analysis of non-redundant metagenome-assembled genomes (MAGs). (A) Phylum-level taxonomic classification of MAGs with Proteobacteria further resolved at class level; (B) Stacked bar plot of MAG genomic novelty across taxonomic ranks (order to species); (C) Phylogenetic tree of 253 non-redundant MAGs. The inner ring’s color scheme represents phylogenetic phyla, the intermediate heatmap shows MAG completeness (%), and the outer bars lengths correspond to genome size.
